# Effects of Transverse Crack on Chloride Ions Diffusion and Steel Bars Corrosion Behavior in Concrete under Electric Acceleration

**DOI:** 10.3390/ma12152481

**Published:** 2019-08-05

**Authors:** Fengyin Du, Zuquan Jin, Chuansheng Xiong, Yong Yu, Junfeng Fan

**Affiliations:** 1 College of Civil Engineering, Qingdao University of Technology, Qingdao 266033, China; 2 Cooperative Innovation Center of Engineering Consrtuction and Safety in Shandong Blue Economic Zone, Qingdao 266033, China; 3 Key Laboratory of Coast Civil Structure Safety, Tianjin University, Ministry of Education, Tianjin 300350, China

**Keywords:** crack width, reinforced concrete, chloride diffusion coefficient, EIS, electrochemical impedance spectroscopy

## Abstract

Cracks greatly impact the durability of concrete structures due to their influence on the migration of chloride ions and the corrosion process of steel bars. This study investigates the effects of transverse cracks on chloride diffusion and the corrosion behavior of two types of steel bars (low carbon steel and corrosion resistant steel) in fly ash concrete with 1 kg/m^3^ solution-polymerized super absorbent polymer. Electrochemical impedance spectroscopy was used to monitor the chloride-induced corrosion behavior of steel bars in concrete. The chloride profile around cracks was tested via chemical titration. The corrosion products diffusion area was photographed and measured to evaluate the influences of cracks on the corrosion degree of steel bars. Transverse cracks greatly influence the chloride ion transport. When their width is less than 0.15 mm, cracks exert little influence on both chloride diffusion and steel corrosion. When the crack width exceeds 0.15 mm, the chloride ion transmission coefficient is significantly improved and steel corrosion is accelerated. However, when the crack width exceeds 0.20 mm, this effect is gradually weakened. Based on the experimental data, a quantitative relationship between the crack width and the chloride ion transmission coefficient in electric acceleration was established.

## 1. Introduction

Reinforced concrete is widely used for marine engineering structures due to its excellent mechanical properties [1], simple construction, and low cost [2]. However, the harsh marine corrosive environment possess a severe challenge to the durability of reinforced concrete [3,4]. Chloride-induced reinforcement corrosion is one of the major threats and leads to the destruction of concrete structures in the marine environment [5].

To extend the service life of marine reinforced concrete structures, the utilized steel bars are often protected by increasing the thickness of the concrete cover which prolongs the ion transportation distance and provides a high alkalinity environment, which forms a thin oxide film on the steel surface [6]. However, due to the effect of load and the inherent defects of concrete itself, many micro-cracks emerge in the concrete cover. Transverse cracks are the most common form of cracks in reinforced concrete structures, and are primarily caused by shrinkage [6], chemical reaction [7], weather processes (e.g., freezing and thawing) [8], and mechanical loading [9]. These cracks provide direct channels for chloride ions and oxygen to enter the concrete structure, thus greatly increasing the risk of corrosion damage to the reinforced concrete structure [10,11,12,13]. Therefore, studying the influences of cracks on the ion diffusion and steel corrosion in concrete is of great significance for the prediction of the durability and service life of marine concrete structures.

Until now, a considerable number of experimental and theoretical studies have investigated the influences of cracks on the durability of cement-based materials, studying e.g., chloride ingress [14,15], carbonation [16,17], and steel corrosion in concrete structures [18,19,20]. Djerbi et al. [21] showed that the chloride ion diffusion coefficient of concrete increases by an order of magnitude in cracked concrete. Berrocal et al. [22] reported that corrosion initiates almost immediately when a wide surface crack is present. In addition, the crack width exerts an important influence on the transport behavior of chloride ions in concrete. This has already been reported by many researchers. For example, Ismail et al. [23] presented an experimental study showing that the threshold crack width is 30 μm. Jang [24] reported that the threshold crack width of steady-state migration for different concrete samples is around 55–80 μm. The critical value of Jang is similar to that of Djerbi et al. [21], which is 80 μm. Therefore, it is clear that the critical crack width is a key indicator of how the transport of ions in concrete is affected. When the width is less than this critical crack width, the crack exerts little effect on the durability of the concrete structure. However, if the crack width exceeds the critical value, the durability of the concrete will be greatly reduced. Although much research has investigated the effect of crack width on concrete, few studies have investigated the influences of the crack width on the entire life time of concrete structures (including chloride ions ingress and steel corrosion process). Ascertaining the quantitative relationship between crack width and concrete durability is significantly important to engineering practitioners. In this paper, the effects of transverse cracks on chloride diffusion and the corrosion behavior of two types of steel bars (low carbon steel and corrosion resistant steel) in fly ash concrete are investigated.

The lack of corrosion resistance of carbon steel is a further primary cause of corrosion damage in reinforced concrete exposed to a chloride containing environment. Hurley et al. [25,26] showed that stainless steel bars offered excellent improvement in corrosion resistance compared to conventional carbon steel bars. However, due to the required addition of alloying elements (12% or more Cr content [27]), stainless steel bars usually have poor weldability, which induces great difficulties for the construction as well as leads to high production cost (about 5–8 times the of ordinary carbon steels) [28]. Moreover, Zheng et al. [29] studied the chloride ion depassivation of passive film on galvanized steel bars in concrete pore solutions. The results showed that the critical chloride value for this type of steel is higher than for ordinary steel bars, implying a better anti-corrosion property of galvanized steel bars. However, galvanized steel bars also have poor machinability and the cladding films crack easily, which causes corrosion; therefore, galvanized steel bars are not suitable for practical engineering. An increasing number of countries are committed to apply low-cost, high-performance corrosion-resistant steels with lower alloying elements. Furthermore, it is rather significant and valuable to investigate the corrosion process induced by cracks to predict future developments and changes. For comparison, the corrosion process of two types of reinforcing steel (low carbon (LC) steel bars and corrosion resistant (CR) steel bars) in concrete are studied under different crack conditions.

In this study, different crack widths are prefabbed and quantitatively investigated in artificially cracked reinforcement concrete after the degenerative process. Reinforced concretes with cracks are prepared and chloride migration is induced via electric acceleration. The chloride-induced corrosion behavior of reinforced steel in concrete is studied via electrochemical impedance spectroscopy (EIS). The effects of crack width and steel types are analyzed, which can help to understand the damage process in reinforced concrete.

## 2. Experiment

### 2.1. Materials and Mix Proportions

P·I.52.5 Portland cement was used as the main cement-based material. Class I fly ash (Chinese standard GB1596-2005 [30]) and S95 granulated blast furnace slag (GGBS, Chinese standard GB/T18046-2008 [31]) were used to partly replace Portland cement. Crushed granite (with a maximum size of 20 mm) was used as the coarse aggregate, and river sand (with fineness modulus of 2.6) was used as the fine aggregate. A polycarboxylic superplasticizer was used, the dosage of which was adjusted to keep the slump of fresh concrete in the range of 180 mm to 220 mm. An air-entraining agent was used, the dosage of which was adjusted to keep the air content of fresh concrete in the range of 3–5%. Solution-polymerized super absorbent polymer (SAP) with irregular particle shape and particle sizes <63 μm in the dry state was used as the internal curing agent. The optimized mixture LF50 was mixed with about 32% GGBS and 17% fly ash and had w/c = 0.35. This was used to line the concrete structure of the Jiaozhou subsea tunnel [1]. The dosage of dry SAP was 1 kg/m^3^ and was first dissolved in 30 g water. The mixture proportions of concretes are listed in Table 1. The chemical composites of both types of reinforcement bars are listed in Table 2.

### 2.2. Preparation of Concrete Samples with Cracks

Reinforced concrete specimens with a size of 100 mm × 100 mm × 300 mm for each mix proportion were cast in the laboratory. Grooves with a depth of 15 mm were precast on the surface of reinforced concrete specimens. Round low carbon (LC) steel bars (10 mm diameter) and deformed CR steel bars (12 mm diameter) were set in concretes with a cover of 25 mm. The surface of the steel bars was polished with 200# sand paper. The steel bars were degreased with acetone prior to being placed in the mold. The effective exposure length of the steel bar was 250 mm.

Plastic flakes with 0.10 mm, 0.15 mm, 0.20 mm, and 0.30 mm thickness were used to prepare transverse cracks perpendicular to the rebar. These plastic flakes were inserted into concrete samples during the initial setting stage and were pulled out before the final setting.

All concrete samples were cured with mold at room temperature for 48 h, and were then stripped and cured at T = 20 ± 3 °C and RH = 95% for 28 days.

### 2.3. Accelerated Corrosion Tests

**Figure 1:**
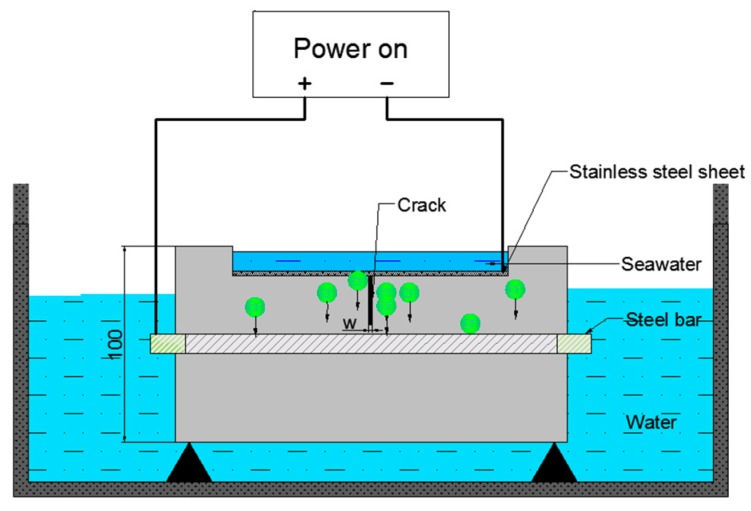
Diagram of potentiostatic accelerated corrosion of reinforced concrete.

A constant voltage of 30 V was applied between the steel bars and negative electrodes to accelerate the diffusion of chloride ions and the corrosion of steel bars. The current was continually determined every 1–2 h during day time and every 8–10 h at night. The entire duration of the accelerated corrosion test was conducted from 100 to 400 h according to different series. Figure 1 shows the diagram of the test. Noteworthy, corrosive ions migrate from the protective cover to the steel surface under constant potential acceleration, and the upper surface of the steel bars was corroded first, which is closer to natural corrosion.

### 2.4. EIS Tests

EIS is a suitable technique to gain information about steel corrosion in concrete by analyzing the corresponding equivalent circuits [32,33,34]. All electrochemical measurements were conducted at room temperature (25 ± 3 °C) with a three-electrode system. The reinforcement steel electrode, Pt foil, and saturated calomel electrode (SCE) were used as the working electrode, counter electrode, and reference electrode, respectively.

### 2.5. Chemical Titration of Chloride Ions

**Figure 2:**
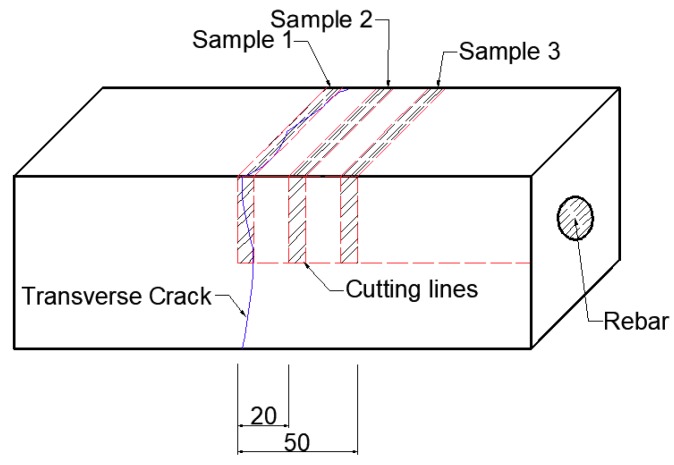
Sampling areas used for the chemical titration of chloride ions (mm).

To determine the influence of cracks on the diffusion of chloride, three different positions from the crack (0 mm, 20 mm, and 50 mm) were extracted to test the chloride content, as shown in Figure 2. Powder samples were taken from the exposed surface at 3 mm per depth. The crack widths are 0.10 mm, 0.15 mm, 0.20 mm, and 0.30 mm, respectively. The three samples are 0 mm, 20 mm, and 50 mm parallel to the crack. The three sampling areas are marked with dotted lines in Figure 2.

The free chloride content was obtained via silver nitration titration (SNT) based on the operations of Testing Code of Concrete for Port and WaterwayEngineering (JTJ 270-98) [35,36].

## 3. Results and Discussion

### 3.1. Chloride Ion Profile

**Figure 3:**
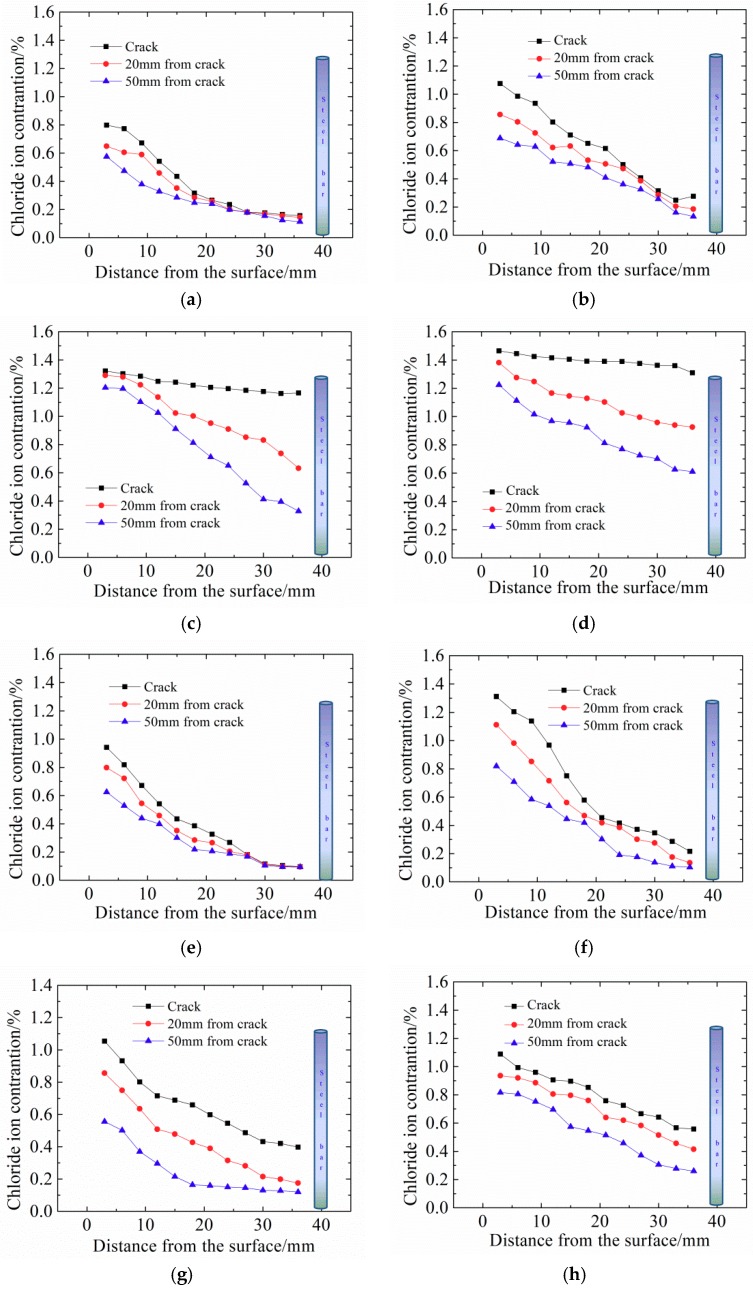
Chloride ion content distribution after electric acceleration. (**a**) Crack width (LC) = 0.10 mm; (**b**) Crack width (LC) = 0.15 mm; (**c**) Crack width (LC) = 0.20 mm; (**d**) Crack width (LC) = 0.30 mm; (**e**) Crack width (CR) = 0.10 mm; (**f**) Crack width (CR) = 0.15 mm; (**g**) Crack width (CR) = 0.20 mm; (**h**) Crack width (CR) = 0.30 mm.

All concrete samples with different crack widths were immersed in seawater for 30 h for electric accelerated corrosion tests. The chloride distributions were tested in concrete with different crack widths and different positions and the results are shown in Figure 3.

Figure 3 shows that the chloride ion content in cracked concrete decreased with depth. For pre-set crack widths of 0.10 mm and 0.15 mm (in Figure 3a,b), the cracks exerted little effect on chloride transport primarily due to the self-healing ability of concrete. For crack widths of 0.20 mm and 0.30 mm, the chloride ion content increased significantly.

The chloride ion content near the crack was clearly higher than at a distance from the crack, because cracks accelerate the transport of chloride ions. The chloride ion content at the crack exceeded that at 20 mm away from the crack, followed by that at 50 mm. However, compared with the chloride content in the crack area, the content 50 mm away from the crack was relatively low, which means that the effects of the crack on chloride transport was not a one-dimensional transport process. The further away from the crack, the smaller the influence of the crack on the chloride ion transport. Therefore, the chloride ion concentration in the cracks (or in the range of 0–20 mm from the crack) is higher than that in the outside area. Jin [16] also reported that the chloride ion content 0–6 mm from the crack was higher than that in the surrounding area; however, that in 6–12 mm was lower than that of the surrounding area. The reason for this is that cracks increase the specific surface area inside the concrete and thus increases the bonding capacity of concrete to chloride ions.

Fick’s second law was applied to fit the chloride ion concentration of concrete with depth and the results are shown in Table 3. The data in Table 3 illustrate that the apparent chloride diffusion coefficient in concrete increases with increasing crack width. When the crack width is 0.30 mm, the diffusion coefficient at the crack increases by two orders of magnitude than that at 0.10 mm. This phenomenon has been found in previous research [23,24]. Ismail [23] explained this reduced diffusion capacity of chloride ions along cracks of around 0.08-0.1 mm by the mechanical interaction between the fracture surfaces where no stress transfer occurs between crack surfaces, which impedes chloride diffusion. Crack widths below 0.15 mm slightly influenced the transmission coefficient 20 mm and 50 mm away from cracks.

**Figure 4:**
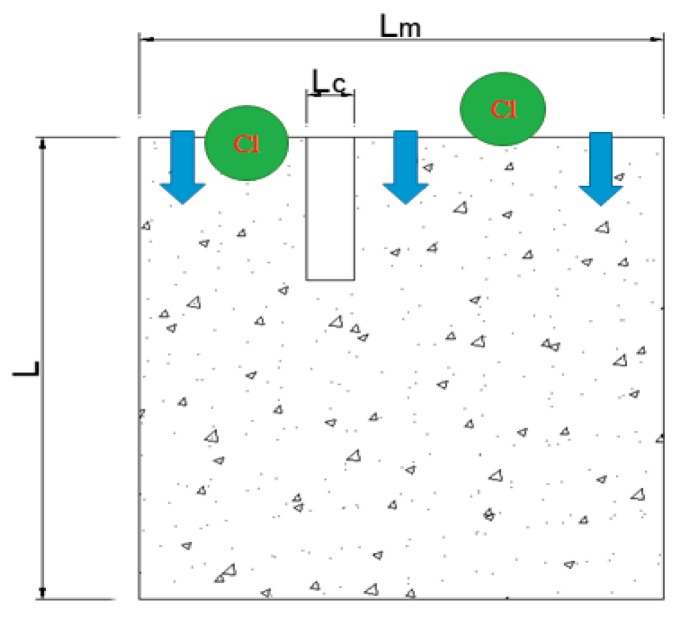
Anisotropic (1-D) cracks in concrete.

The following details the influences of crack width on the diffusion coefficient of chloride ion D [37,38,39]. The crack distribution in a concrete unit is shown in Figure 4 where the crack direction is consistent with the chloride ion diffusion direction.

Figure 4 shows that:

(1) $$A_{m}=L\times L_{m}$$

(2) $$A_{c}=L\times L_{c}$$

where, *L_c_* represents the crack width, *L_m_* represents the length of the concrete unit, *L* represents the height of the concrete unit, *A_c_* represents the crack area, and *A_m_* represents the concrete unit area.

The external ions diffuse into the interior of the concrete samples, and the diffusion power depends on the chemical potential. *F_sum_* represents the chemical potential of the concrete samples. The total chemical potential *F_sum_* should be equal to the sum of the chemical potential *F_c_* that drives the chloride ions toward cracks and the chemical potential *F_s_* that drives the chloride ions toward the matrix:

(3) $$F_{\mathrm{sum}}=F_{c}+F_{m}$$

The diffusion flux of the concrete unit microelement equals the sum of the components divided by the total area:

(4) $$j_{\mathrm{sum}}=\frac{j_{c}\times A_{c}+j_{m}\times A_{m}}{A_{c}+A_{m}}$$

where, *j_sum_*, *j_c_*, and *j_m_* represent the sum of diffusion flux, the diffusion flux in the crack, and the diffusion flux of concrete without cracks, respectively (mol/(m^2^·s)).

The diffusion flux is equal to the diffusion chemical potential energy multiplied by the diffusion coefficient:

(5) $$j_{\mathrm{sum}}=-D_{\mathrm{sum}}\times F_{\mathrm{sum}}$$

(6) $$j_{c}=-D_{c}\times F_{c}$$

(7) $$j_{m}=-D_{m}\times F_{m}$$

Based on the above three formulas, the relationship between the overall diffusion coefficient and the crack and matrix diffusion coefficient can be obtained as:

(8) $$D=\frac{D_{c}\times A_{c}+D_{m}\times A_{m}}{A_{c}+A_{m}}$$

Combining Equations (1) and (2), the relationship between the chloride ion diffusion coefficient of cracked concrete and the collective chloride ion diffusion coefficient is:

(9) $$D=\left(1+\frac{D_{c}\times L_{c}}{D_{m}\times L_{m}}\right)\times D_{m}$$

where, *D* represents the diffusion coefficient of chloride ions in concrete with cracks; *D_m_* represents the diffusion coefficient of chloride ions in concrete without cracks; *D_c_* represents the diffusion coefficient of chloride ions in cracks;

However, cracks influence the chloride diffusion coefficient. According to the experimental results, it is advisable to use the chloride ion diffusion coefficient at the crack of 0.30 mm as a constant reference value. Hence, the diffusion coefficient of chloride ions in cracks can be expressed as:

(10) $$D_{c}=\frac{D_{e}}{1+\mu }$$

where, *D_e_* represents the chloride diffusion at a crack of 0.30 mm in an electric field, 2.17 × 10^−2^ m^2^/s; *D_c_* represents the chloride diffusion in the crack in an electric field; μ represents the resistance of cracks to chloride ions.

Therefore, the relationship between the diffusion of chloride ions and the crack width is:

(11) $$D=\left(1+\frac{D_{e}\times L_{c}}{\left(1+\mu \right)\times D_{m}\times L_{m}}\right)\times D_{m}$$

**Figure 5:**
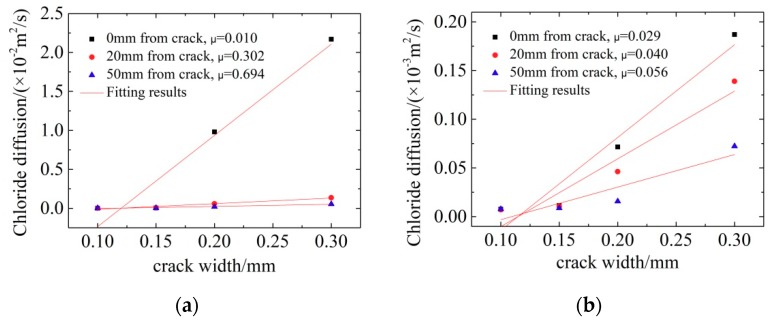
Relationship between diffusion coefficients and distance from crack. (**a**) Concrete with LC; (**b**) Concrete with CR.

According to the Equation (8), the apparent chloride diffusion coefficient of concrete with different crack widths can be calculated and compared with the test results. Figure 5 shows the results.

Figure 5 and Equation (11) can simulate the relationship between the chloride ion diffusion coefficient and the crack width in the cracked concrete. Clearly, with increasing crack width, the chloride ion diffusion coefficient increases linearly both at the crack and in the peripheral region of the crack. In addition, the resistance of the crack in chloride ion transport in cracks is significantly smaller than in the surrounding areas.

When the crack width is less than 0.15 mm, the resistance μ is relatively high, which indicates that the cracks impose a strong inhibitory effect on the transport of chloride ions. However, when cracks are wider than 0.15 mm, chloride ions have more space to invade the interior of concrete, and consequently, the μ value is much smaller. Similar results have also been obtained by other researchers [14,40,41]. Jang [24] reported that the diffusion coefficients showed an increase at a crack width of around 0.20 mm. According to studies of Wang et al. [14] and Gagne [40], the “threshold crack width” for diffusion was 0.015 mm and 0.055 mm, respectively. This discrepancy was possibly due to the inaccuracy in the measurement of current density or initial chloride concentration.

Moreover, the diffusion coefficient of concrete with distances of 20 mm and 50 mm showed similar results, which means that the affecting scope of cracks on the diffusion coefficient of concrete is less than 20 mm.

### 3.2. EIS Measurements for Different Corrosion Times and Crack Widths

#### 3.2.1. LC Steels

**Figure 6:**
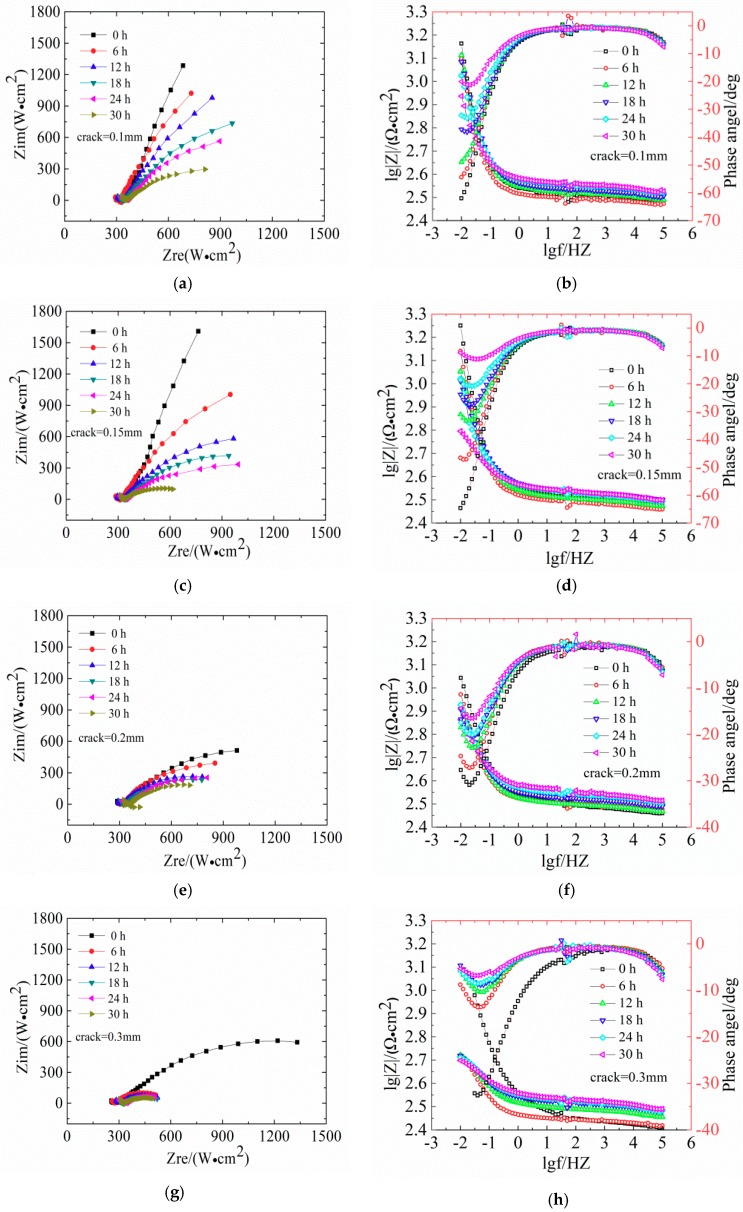
Evolutions of Nyquist plots and bode diagrams for reinforcement low carbon (LC) concrete with different crack widths. (**a**,**b**) Crack width = 0.10 mm; (**c**,**d**) Crack width = 0.15 mm; (**e**,**f**) Crack width = 0.20 mm; (**g**,**h**) Crack width = 0.30 mm.

Figure 6 shows the evaluation of Nysquist plots and the Bode diagrams of LC steels in concrete samples.

Figure 6 shows the EIS diagrams of reinforcement steel specimens after 30 h of immersion in seawater with different crack widths (Nyquist plots and Bode diagrams). The Nyquist plots show a diameter of the capacitive loop, which reflects the resistance on the surface of the steels. The corrosion of steel in low-frequency reaction concrete in the impedance spectrum is mainly controlled by the material transfer process. In the Nyquist diagram, the slope of the curve in the low frequency region increases, indicating that the transmission resistance is large, which means that the steel bars are in a passivated state. With increasing exposure age, the capacitive arc is present in the low frequency region, and the radius of the capacitive arc is smaller; therefore, corrosive resistance is reduced. In the Bode phase, a larger absolute value of the maximum phase angle in the low frequency region indicates a more stable metal in the electrochemical system.

The diameter of the capacitive loop shrunk gradually with exposure time. In Figure 6a,c, the Nyquist plots of reinforced concrete with crack widths of 0.10 mm and 0.15 mm show slight deviations. This indicates that the impedance gradually increased and the reinforced concrete was gradually compacting. The slope of low-frequency curve is not obvious, indicating that the high-alkaline environment exerts a beneficial effect on the steel bar passivation. Figure 6e shows that the Nyquist plots of concrete with 0.20 mm shows a significant capacitive reactance arc, indicating the degeneration of steel bars. The Bode diagram in Figure 6f shows that the absolute value of the maximum phase angle in the low-frequency area decreases from 31° at 0 h to 16° at 30 h, indicating the destruction of the steel passivation film. Figure 6h shows that the capacitive reactance arc radius in Nyquist plots of the concrete with 0.30 mm is strongly decreasing and the absolute value of the maximum phase angle is 7° at 30 h, indicating the depassivation of the steel bars.

The Nyquist and Bode phase plots were only qualitatively analyzed by the graph topological structure of the corrosion of the steel bars. Therefore, the future parameters were obtained through the fitting software Zsimpwin and Stern–Geary formula. The software Zsimpin was used to fit impedance spectroscopy to obtain *R_p_* and Equation (12) [41] was used to calculate the corrosion current density *i_corr_*.

**Figure 7:**
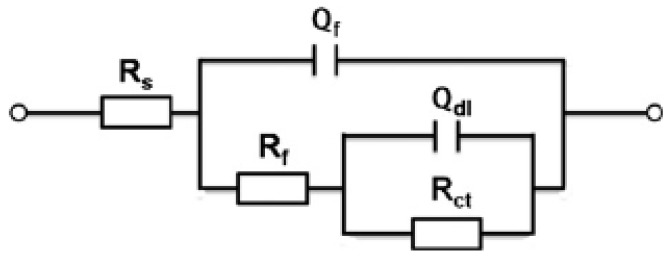
Equivalent circuit applied to simulate the electrochemical impedance spectroscopy results for reinforcement steel.

According to previous research [41,42,43], equivalent circuits with two-time constant are generally selected for the fitting of reinforced concrete. As shown in Figure 7, the R{R[Q(R(QW))]} equivalent circuit was selected in this experiment [41,42]. In this equivalent circuit, the passive film is considered to have a porous structure and show non-ideal capacitive behavior [43]. *Rs* represents the resistance of the simulated solution, *Rct* represents the charge transfer resistance of the utilized steel, *Q_dl_* represents the double laver capacitance at the steel-solution interface, *R_f_* and *Q_f_* represent the resistance and capacitance of the passive film, respectively. The constant phase element (CPE, expressed as *Q*) was used as an alternative to the pure capacitor to represent non-homogeneity of the steel surface.

EIS measurement was conducted in the frequency range of 10^−2^–10^5^ Hz and an AC signal with an amplitude of 10 mV. The corrosion current density (*i_corr_*) was based on the Stern–Geary relationship:

(12) $$i_{\mathrm{corr}}=\frac{B}{R_{p}}$$

where, *R_p_* represents the polarization resistance and *B* is a constant as a function of anodic and cathodic Tafel slopes. For steels in concrete, the *B* value is normally equal to 26 mV for active state and 52 mV for passive state [32,44,45]. In this work, a value of 26 mV was used to calculate the corrosion.

**Figure 8:**
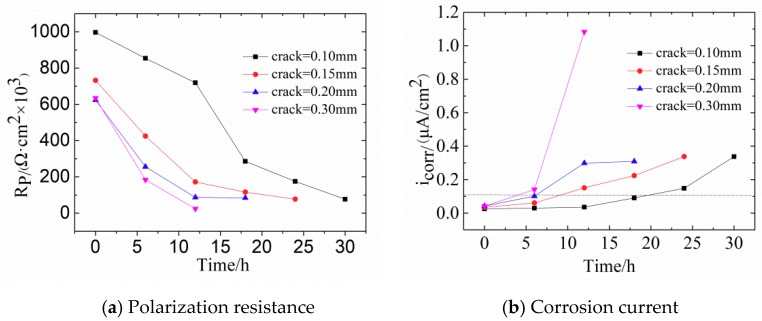
(**a**) Polarization resistance and (**b**) corrosion current density for concrete reinforced with low carbon steel with different crack widths.

Figure 8 shows the changes of *R_p_* and *i_corr_* in concrete with different crack widths. With increasing width of transverse cracks, *R_p_* becomes smaller. For crack widths of 0.10 mm and 0.15 mm, *R_p_* is relatively large, which means that the fine cracks slightly influence steel corrosion. Noteworthy, the value of *R_p_* changed slightly when the corrosive time was shorter than 12 h, indicating that steel bars are in the passive stage. The possible reason for this phenomenon is the self-healing of concretes with fine cracks. The main causes of this are: (1) The volcanic ash effect of fly ash produces Ca(OH)_2_ and CSH gel, which exert a certain filling effect on internal micro-cracks [46]; (2) planktonic microorganisms and fine sand particles in the seawater block the surface cracks so that oxygen cannot reach the surface of steel bars within a short time.

Figure 8b shows that the corrosion current density follows *i_corr0.30_* > *i_corr0.20_* > *i_corr0.15_* > *i_corr0.10_*. For crack widths of 0.10 mm and 0.15 mm, more than 8 h are needed for the corrosion of steel bars. According to Table 4, when *i_corr_* exceeds 0.1 μA/cm^2^, steel bars enter the low-speed corrosion stage.

The current density of steel bars is an important chemical parameter; however, it is difficult to evaluate the degree of corrosion in reinforced concrete. Therefore, it needs to be converted into the corrosion rate of steel bars. According to Faraday’s law, the corrosion velocity can be expressed as:

(13) $$R_{p}=\gamma \cdot t+R_{0}$$

(14) $$H=\frac{M\cdot i_{\mathrm{corr}}\cdot t}{\rho \cdot n\cdot F}$$

where, γ represents the rate of polarization resistance values change; *t* = time, h; *R*_0_ represents the initial polarization resistance, KΩ·cm^2^; *H* represents the corrosive depth, μm/a; *M* represents the molar mass of Fe, 56 g/mol; *i_corr_* represents the corrosive current density, μA/cm^2^; *t* represents the corrosion time, s; ρ represents the density of Fe, 7.9 × 10^3^ kg/m^3^; *n* represents the charge of reactive ion, (Fe→Fe^2+^ + e^−^); *F* is the Faraday constant, 96,485 C/mol. This formula shows that 0.1 μA/cm^2^ corrosive current density leads to 1.587 × 10^−3^ μm loss depth of steel bars per 12 h.

Table 5 shows that the initial *R_p_* maintains around 600–1000 KΩ·cm^2^. With increasing crack width, the rate of *R_p_* change gradually increases, which indicates that the larger crack width leads to a greater corrosion risk of steel bars. For a crack width of 0.10 mm and 0.15 mm, 19 h and 9 h are respectively needed for steels to enter the low-rate corrosion condition. With the increasing crack width and corrosion rate, the corrosion rates of steel bars in concrete with crack widths of 0.10 mm and 0.15 mm were 0.574 × 10^−3^ μm/12 h and 2.399 × 10^−3^ μm/12 h, respectively, compared to that of reinforced concrete with 0.30 mm crack width where corrosion rate was 17.136 × 10^−3^ μm/12 h, indicating a severe corrosion condition.

#### 3.2.2. Corrosion Resistant Steel Bar

**Figure 9:**
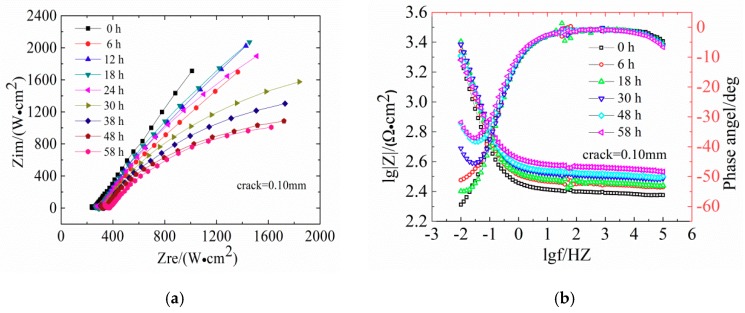

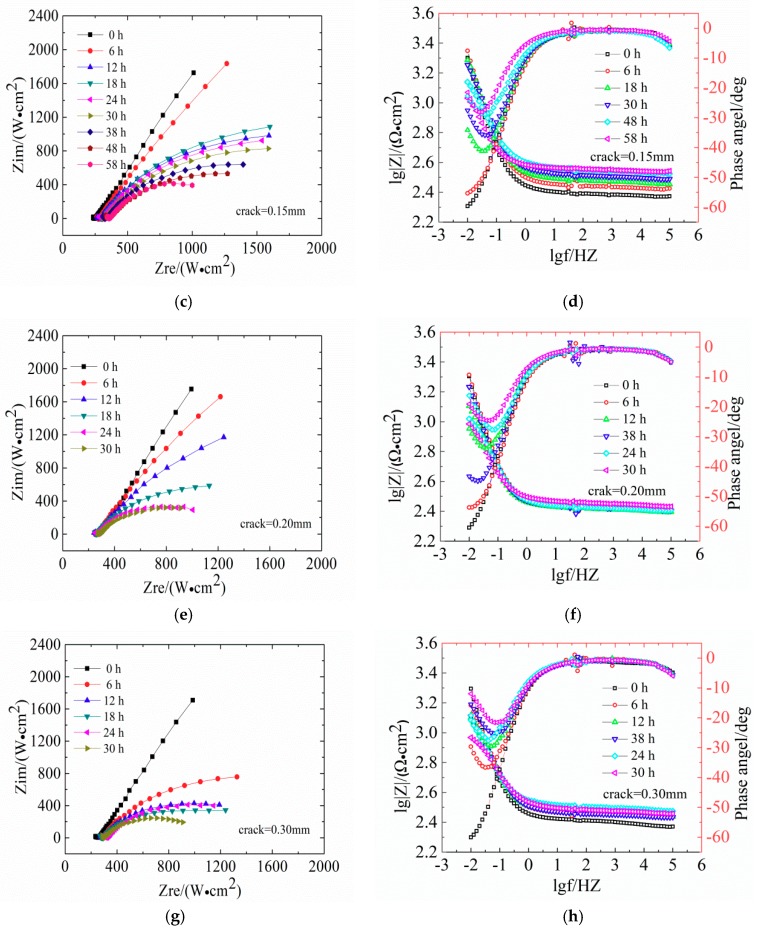
Evolutions of Nyquist plots for reinforced corrosion resistant concrete with different transverse crack widths. (**a**,**b**) Crack width = 0.10 mm; (**c**,**d**) Crack width = 0.15 mm; (**e**,**f**) Crack width = 0.20 mm; (**g**,**h**) Crack width = 0.30 mm.

The same experiment was conducted for reinforced concrete specimens with corrosion resistant steel bars. The evolutions of Nyquist plots and the Bode diagrams of CR are shown in Figure 9.

**Figure 10:**
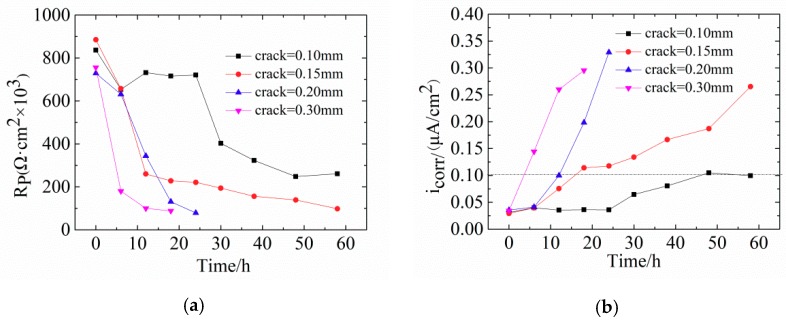
(**a**) Polarization resistance and (**b**) corrosion current density for reinforced corrosion resistant concrete with different transverse crack widths.

Comparison of the Nyquist plots between Figure 7 and Figure 9 clearly shows that the reduction of the capacitive arc radius of CR steel bars in concretes is significantly smaller than that of LC steel bars, indicating that the failure rate of the passivation film of the LC steels is faster than that of CR steels. Moreover, the Bode diagrams in Figure 9 show that the maximum phase angle of concrete with low-frequency area decreased to 6°, indicating severe damage of steel bars. Figure 10 shows the changes of R_p_ and i_corr_ of CR steel bars in concrete.

Comparison between Figure 8 and Figure 10 shows that the reduction rate of the *R_p_* value of CR steel bars is significantly smaller than that of LC steel bars. The smaller the crack width is, the slower the reduction rate will be. When the crack width is 0.10 mm, 48 h and 19 h are needed for CR steels and LC steels, respectively, indicating that CR steels have better corrosion resistance than LC steels. Figure 10 shows that the time for concretes with 0.30 mm crack widths to be eroded is much shorter than that with 0.10 mm (4 h and 48 h, respectively). When the accelerated time was 12 h, the corrosive current density of concrete with 0.30 mm crack was 0.30 μA/cm^2^.

The absolute value of the slope of *R_p_* of LC steels exceeds that of CR steels and the smaller the crack width is, the slower the slope change. In addition, for a crack width of 0.30 mm, the corrosion rate of CR steels is much smaller than that of LC steels, at 4.126 × 10^−3^ μm/12 h and 17.136 × 10^−3^ μm/12 h. Furthermore, the better corrosion resistance of CR steels is also indicated by Table 6.

### 3.3. Corrosion Production Distriubtion Area

**Figure 11:**
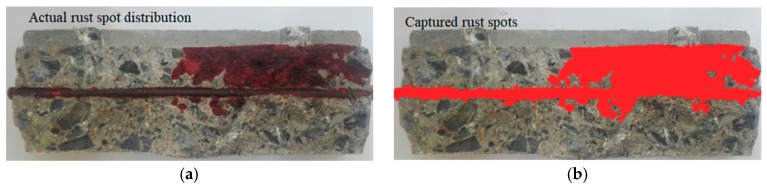
Corrosion analysis of corroded reinforced concrete. (**a**) Actual rust spot distribution; (**b**) Captured rust spots.

To quantitatively analyze the relationship between crack width and corrosion production distribution area the percent of the original area in reinforced concrete was used. The corroded area can be calculated by the software Image-Pro Plus. This software automatically captures the gray value of each pixel of the acquired image and calculates the gray standard deviation of the image. The calculation formula is shown in Equation (15). The gray value represents the different brightness levels of the image. It is useful to darken the rust area to improve accuracy, as shown in Figure 11.

(15) $$\mathrm{std}=\sqrt{\frac{\sum_{t=1}^{M}\sum_{j=1}^{N}{\left(Gray\left(i,j\right)-\bar{Gray}\right)}^{2}}{M\times N}}$$

where, Std represents the image gray standard deviation, **M** × **N** represents the two dimensional matrix, *Gray(i, j)* represents the gray level of each pixel in the image, and $\bar{\mathrm{Gray}}$ represents the average gray level.

**Figure 12:**
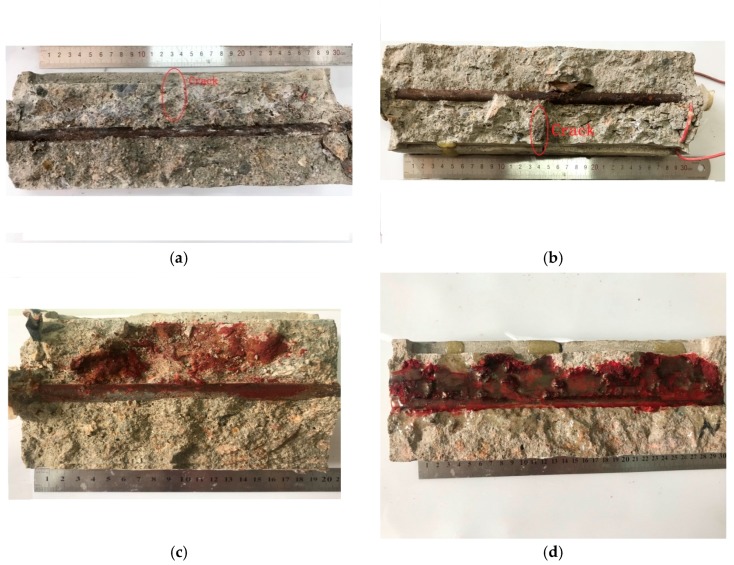
Pictures of concrete samples (LC) with transverse crack widths. (**a**) Crack width (LC) = 0.10 mm; (**b**) Crack width (LC) = 0.15 mm; (**c**) Crack width (LC) = 0.20 mm; (**d**) Crack width (LC) = 0.30 mm.

**Figure 13:**
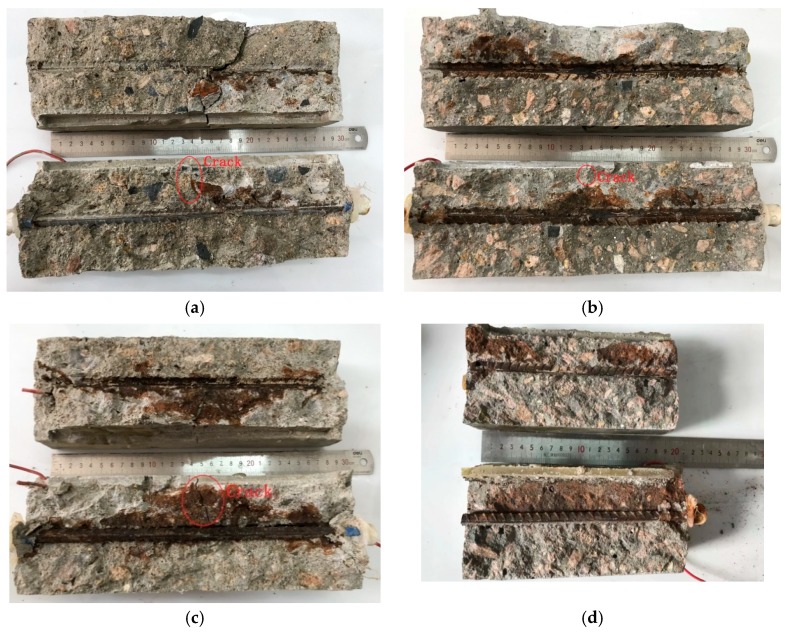
Corrosion production distribution area of concretes with CR steels. (**a**) Crack width (LC) = 0.10 mm; (**b**) Crack width (LC) = 0.15 mm; (**c**) Crack width (LC) = 0.20 mm; (**d**) Crack width (LC) = 0.30 mm.

The results of prying the test piece are shown in the Figure 12 and Figure 13. The corrosion products of reinforced concrete were distributed in the vicinity of the protective cover. This proved that the single-surface osmosis corrosion solution using the constant potential acceleration matches the natural corrosion and thus, the accelerated corrosion method is feasible.

Table 7 shows the calculated corrosion production distribution area *S* (%) and crack width *w* (mm) of different concrete obtained from the experiments.

Reinforced concretes with 0.10 mm, 0.15 mm, 0.20 mm, and 0.30 mm cracks have different corrosion production distribution areas. Accelerated corrosion is an uneven corrosion and the wider the crack width, the larger the corrosion production distribution area. When the crack width remains below 0.15 mm, the corrosion production distribution area *S* is ~10%, which means that fine crack widths only slightly influence the corrosion production distribution area due to self-healing. However, the corrosion production distribution area in concrete with 0.30 mm crack width is almost four to five times as high as that in concrete with 0.15 mm crack width. This indicates that wider cracks (>0.15 mm) exert strong influence on concrete corrosion, which has also been reported before [14,48]. Therefore, crack widths larger than 0.15 mm exert a large influence on the corrosion of reinforced concrete.

**Figure 14:**
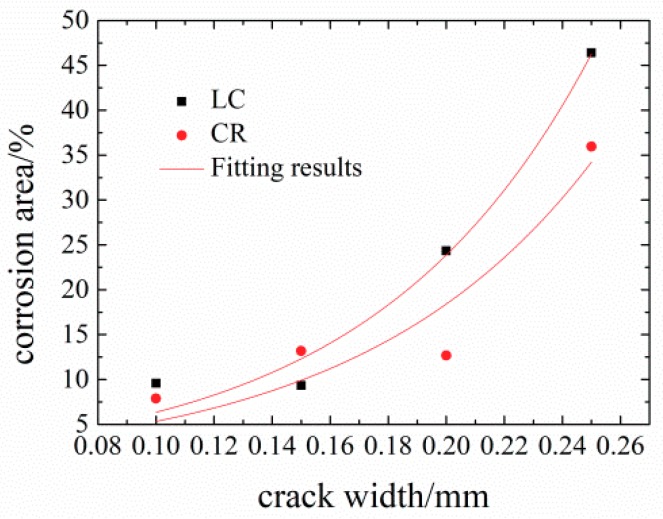
Fitting results of crack width and corrosion production distribution area.

According to previous research [10], the liner relationship between the crack width and the corrosion production distribution area was established as $W_{s}=39.14\rho -0.0494$, $W_{s}=\sum_{k-1}^{j}w_{sk}$, where *W_s_* represents the sum of all crack widths along the top and side of the slice. However, this model does not match the experimentally obtained results. Figure 14 shows the relationship between the corrosion production distribution area *S* and crack width *w*.

*S*_LC_ = e^8.2*x*−1.3^ (R^2^ = 0.978), (16)

*S*_CR_ = e^8.0*x*−1.2^ (R^2^ = 0.878) (17)

Clearly, the derivative of the regression curves has an inflection point at 0.15 mm. This indicates that when the crack width is below 0.15 mm, the corrosion area is less affected by cracks. Once the crack width exceeds 0.15 mm, the corrosion production distribution area *S* is exponentially influenced by crack width as shown in Figure 14, which agrees with the test results.

## 4. Conclusions

In this paper, transverse cracks with 0.10 mm, 0.15 mm, 0.20 mm, and 0.30 mm width were prepared to study the chloride diffusion and corrosion behavior in fly ash concrete, reinforced with two types of steel bars (LC and CR) using the electro-chemical technology. Transverse cracks impact the migration of chloride ions. Crack widths smaller than 0.15 mm impose little influence on chloride ions transmission due to the self-healing effect inside the cracks. For crack widths of 0.15 mm and 0.20 mm, the chloride ion migration coefficient increased by one to two orders of magnitude and steel corrosion is accelerated. However, for crack widths exceeding 0.20 mm, this effect gradually weakened. The effect range of transverse cracks on chloride ion transport is ~20 mm. The chloride migration coefficient 20–50 mm from the cracks is similar to that of more than 50 mm from cracks. Transverse cracks affect the steel corrosion process by accelerating the corrosion rate of steel bars and increasing the corrosion production distribution area. Wider crack leads to a faster corrosion rate. The corrosion rate and corrosion production distribution area in concrete with crack of 0.30 mm is almost four times as much as that in concretes with 0.15 mm. CR steels can be used in reinforced concrete structures in marine environments because the corrosion-resistance of CR steel is superior to that of LC steels due to the naturally forming passive film in low alloy steels.

## Figures and Tables

**Table 1:** Mix proportions of concretes and their properties.

No.	kg/m^3^						Compressive Strength (MPa)		Air Content of Fresh Concrete (%)
	Cement	GGBS	Fly Ash	Sand	Aggregate	SAP with Water	7d	28d	
LF50SP1	250	145	75	730	1095	30	52.6	61.5	3.5

**Table 2:** Chemical compositions of steel bars (%).

Type	The Chemical Compositions								
	Fe	C	Si	Mn	P	S	V	Cr	Mo
CR	Bal.	0.01	0.49	1.49	0.01	0.01	0.06	10.36	1.16
LC	Bal.	0.22	0.53	1.44	0.02	0.02	0.04	-	-

**Table 3:** Apparent chloride diffusion coefficients in different concrete samples with transverse cracks (m^2^/s).

No.	Distance from the Crack		
	0 mm	20 mm	50 mm
LC-0.10 mm	1.72 × 10^−5^	1.98 × 10^−5^	2.10 × 10^−5^
LC-0.15 mm	4.21 × 10^−5^	4.64 × 10^−5^	4.54 × 10^−5^
LC-0.20 mm	9.80 × 10^−3^	5.88 × 10^−4^	2.05 × 10^−4^
LC-0.30 mm	2.17 × 10^−2^	1.36 × 10^−3^	5.40 × 10^−4^
CR-0.10 mm	7.66 × 10^−6^	7.01 × 10^−6^	7.81 × 10^−6^
CR-0.15 mm	1.13 × 10^−5^	1.04 × 10^−5^	8.82 × 10^−6^
CR-0.20 mm	7.15 × 10^−5^	4.62 × 10^−5^	1.57 × 10^−5^
CR-0.30 mm	1.87 × 10^−4^	1.39 × 10^−4^	7.23 × 10^−5^

**Table 4:** Recommended standard for corrosion current density of reinforcement in concrete [47].

Corrosion Stage	*i_corr_* (μA/cm^2^)
Passivation stage	<0.1
Low speed corrosion stage	0.1–0.5
Medium speed corrosion stage	0.5–1
High speed corrosion stage	>1

**Table 5:** Depassivation time and corrosion rate of steel bars in concrete with different crack widths in an electric field.

Crack Width (mm)	γ	*R*_0_ (KΩ·cm^2^)	*R*^2^	t (h)	*H* (×10^−3^ μm/12 h)
0.10	−33.67	997	0.942	19	0.574
0.15	−26.98	732	0.823	9	2.399
0.20	−29.82	624	0.993	6	4.731
0.30	−50.92	635	0.859	4	17.136

**Table 6:** Depassivation time and corrosion rate of steel bars in concrete with different crack widths in an electric field.

Crack Width (mm)	γ	*R*_0_ (KΩ·cm^2^)	*R*^2^	*t*_1_ (h)	*H* (×10^−3^ μm/12 h)
0.10	−10.96	837	0.816	48	0.564
0.15	−11.04	885	0.850	16	1.199
0.20	−30.00	729	0.942	12	1.587
0.30	−34.7	756	0.656	4	4.126

**Table 7:** Corrosion area *S* of different concretes (%).

No.	Corrosion Production Distribution Area S/%
LC-0.10 mm	9.58
LC-0.15 mm	9.35
LC-0.20 mm	24.34
LC-0.30 mm	46.40
CR-0.10 mm	9.59
CR-0.15 mm	13.17
CR-0.20 mm	24.34
CR-0.30 mm	35.96
